# Autophagy: An Important Biological Process That Protects Plants from Stressful Environments

**DOI:** 10.3389/fpls.2016.02030

**Published:** 2017-01-11

**Authors:** Wenyi Wang, Mengyun Xu, Guoping Wang, Gad Galili

**Affiliations:** ^1^Department of Plant Science, Weizmann Institute of ScienceRehovot, Israel; ^2^College of Horticulture, South China Agricultural UniversityGuangzhou, China

**Keywords:** autophagy, protein degradation, abiotic stress, nbr1, TPSO, ATI1

Plants are sessile organisms that cannot escape from stressful environments, such as drought, high salinity, high temperature, and shortage of essential minerals in the soil. Hence, plants have evolved processes that protect them from these harmful conditions. One of these major processes is autophagy (which means, “self-eating”), a mechanism that destroys specific compounds that participate in efficient growth and requires extensive energy input and on the other hand stimulates biological processes that protects from the stress. Autophagy can be either a bulk process, turning over bulk amounts of various components in response to major stresses, such as serious accumulation of damaging compounds in the soil, or a selective process turning over specific components in response to specific and/or relatively minor environmental cues, such as minor shortage of rain and/or non-significant shortage of minerals in the soil (Han et al., 2011; Avin-Wittenberg et al., 2012; Liu and Bassham, 2012; Michaeli et al., 2016).

## The autophagy-related genes (*ATG*s) involved in abiotic stress in plants

The identification of autophagy-related genes (*ATG*s) was an important milestone in the understanding of the mechanism of autophagy. Thus far, over 30 *ATG*s have been identified in *Arabidopsis*, rice, tobacco, and pepper based on comprehensive, genome-wide analysis (Xia et al., 2011; Zhou et al., 2015; Zhai et al., 2016). ATGs can be divided into three categories with respect to their function: (i) ATG1-ATG13 comprising the kinase complex, an upstream regulator that initiates autophagosome formation (Suttangkakul et al., 2011); (ii) The ATG9 and ATG6/vps30 complexes involved in vacuolar protein sorting in which ATG9 interacts with ATG2 and ATG18, boosting phagophore expansion. This operates *via* diverse shuttling of endomembranes, such as those of the endoplasmic reticulum (ER), Golgi, and mitochondria (Tooze and Yoshimori, 2010; Yang and Klionsky, 2010; Kang et al., 2011). The ATG6/vps30 complex recruited by ATG14, which localizes to the pre-autophagosomal structure (PAS) as well as the vacuolar membrane, to generate the autophgosomes (Tooze and Yoshimori, 2010); (iii) ubiquitin like conjugation systems (ATG5-ATG12 complex and ATG8-PE complex), which is essential for autophagosome formation. The ATG8–PE complex has been proven to recruit the cytoplasmic cargo to ensure autophagosome maturation and closure, and it is subsequently transported to the vacuole for degradation (Bassham, 2007; Michaeli et al., 2016). Most of complexes participating in each selective autophagy process have been identified in yeast and animal systems. However, it is still in infancy in plants (Michaeli et al., 2016).

Among the *ATG* genes, *ATG8* is the central protein involved in autophagy and also a marker for the autophgosome (Shpilka et al., 2011). It has been shown to participate in various processes, such as diverse intracellular trafficking, post-mitotic Golgi reassembly, cargo receptor recognition, conjugation to phosphatidylethanolamine, etc. (Kwon and Park, 2008; Tooze and Yoshimori, 2010; Yang and Klionsky, 2010). Moreover, *ATG8* plays an important role in the sensitivity of plants to abiotic stresses. There are nine isoforms of *ATG8* in *Arabidopsis* (*ATG8a* to *ATG8i*). The over-expression of *AtATG8f* led transgenic *Arabidopsis* plants to be more sensitive to mild salt and/or osmotic stress. This kind of sensitivity was accompanied by the modification of root architecture (Sláviková et al., 2005). Furthermore, the overexpression of *SiATG8a* improved the tolerance to nitrogen starvation and drought stress in transgenic *Arabidopsis* plants (Li et al., 2015).

The other *ATG*s also play critical roles in the stress response, especially the response to carbon and nitrogen starvation (Han et al., 2011). Two classical autophagy-related mutants, *atg5* and *atg7*, showed hypersensitivity to carbon and nitrogen starvation (Phillips et al., 2008; Yoshimoto et al., 2009). Autophagy-defective RNAi-*AtATG18a* plants displayed enhanced sensitive to salt, drought and methy viologen treatments compared with wild-type plants (Xiong et al., 2007; Liu et al., 2009). Moreover, *atg13* double-knockout (*atg13a atg13b*) and *atg11* knockout plants also showed a classical *atg* mutant phenotype, which exhibited increased sensitivity to carbon and nitrogen starvation (Suttangkakul et al., 2011). Rice *Osatg10b* mutants were sensitive to treatments with high salt (250mM) and methyl viologen (MV) (Shin et al., 2009). Additionally, a series of autophagy-deficient mutants, such as *atg2-1, atg5-1, atg7-3, atg10-1*, were hypersensitive to submergence stress (Chen et al., 2015).

## NBR1-mediated selective autophagy makes the highly ubiquitinated soluble proteins prone to aggregation during abiotic stresses

Out of a number of autophagy cargo receptors, the cargo receptor NBR1 (NEIGHBOR OF BRCA1 GENE 1) is one of the critical components of the autophagy process. NBR1 was identified in yeast, mammals and plants (Johansen and Lamark, 2011). NBR1 was the first selective cargo receptor, which was found to be responsible for sequestration of ubiquitinated proteins to the vacuole for their degradation inside this organelle. However, the involvement of the cargo receptors in autophagy is poorly understood. Recently, functional homologs of NBR1 proteins were identified in *Arabidopsis* (AtNBR1) and tobacco (JOKA2) plants (Svenning et al., 2011; Zientara-Rytter et al., 2011).

**Figure 1:**
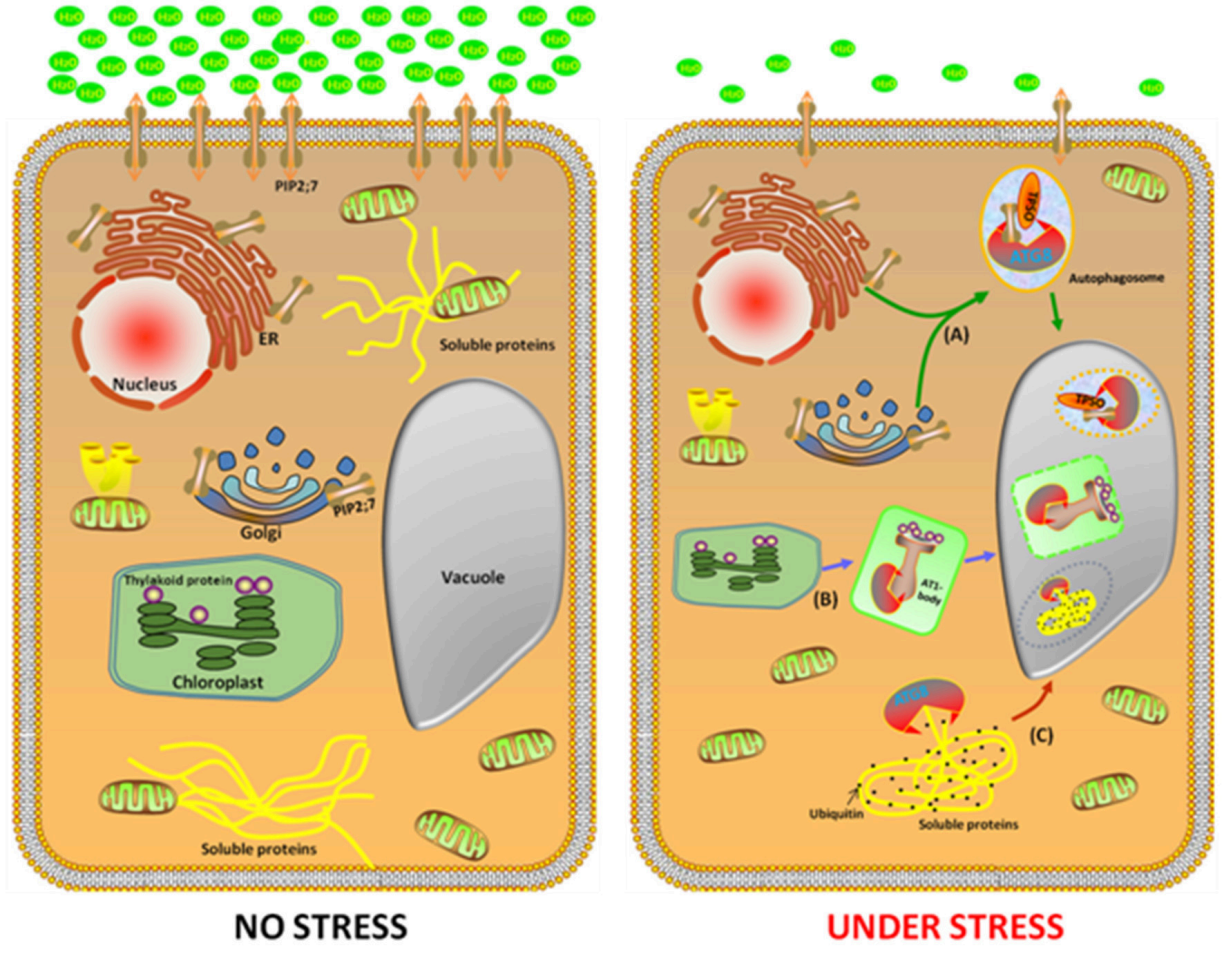
**Identification of three potential cargo receptors in plants**. In the favorable growth conditions (left cell), a large number of genes, such as *NBR1, TPS*O, and *ATI1* were expressed with a low level to maintain homeostasis. Under abiotic stress conditions (right cell), *NBR1, TPSO*, and *ATI1* were induced. In the process of TSPO-mediated selective autophagy pathway, TSPO is transcriptionally upregulated during heat and drought stress, then interacts with PIP2;7 and ATG8 to enable the degradation of porphyrins *via* an autophagy-dependent degradation mechanism **(A)**. The second pathway is ATI1 interact with chloroplast protein in the ATI1-body, then this compound transport their cargo proteins to autophagosomes, which eventually transport to the vacuole for degradation **(B)**. In the NBR1-mediated selective autophagy pathway, an insoluble highly ubiquitinated detergent-resistant protein and ATG8 were shown to be prone to aggregation to form autophgosome, thereby enabling recognition by NBR1 and its subsequent transport to the vacuole for degradation **(C)**.

The *Arabidopsis* NBR1 is a homolog of the mammalian autophagic adaptor NBR1, with an ubiquitin-association domain binds selectively to six of the nine *Arabidopsis* ATG8 protein isoforms. The *nbr1* mutant exhibits sensitivity to a spectrum of abiotic stresses, similar to the autophagy-deficient *atg5* and *atg7* mutants, but had no obvious effect onthe response to carbon starvation and resistance to a necrotrophic fungal pathogen. This indicates that AtNBR1 participates in the response to abiotic stress (Zhou et al., 2013). Under high heat conditions, an insoluble highly ubiquitinated detergent-resistant protein was shown to be prone to aggregation, thereby enabling recognition by NBR1 and its subsequent transport to the vacuole for degradation. Moreover, Rubisco activase and a number of catalases are linked to the response of plants to a wide variety of abiotic stresses and these enzymes accumulated in the *nbr1* mutant (Zhou et al., 2014). These findings suggest that NBR1-mediated selective autophagy pathway plays a critical role during abiotic stresses (Figure 1).

## Abiotic stress-induced TSPO-related protein reduces aquaporin PIP2;7 through autophagic degradation

To decipher the involvement of autophagy in abiotic stress in plants, the identification of the cargo receptor in the process of autophagy is crucial. This is because of the following reasons (i) under normal conditions, autophagy maintains a basal level for homeostasis; (ii) under abiotic stress, cargo receptors are active to remove the damaged or unwanted materials or to recycle the materials for providing anabolic substrates and metabolites to the cells. In plants, one of the critical processes of autophagy is the transport of the unnecessary components to the vacuole for degradation, which is a selective process requiring the cargos.

To minimize the effects of abiotic stresses, plants have developed a sophisticated protein quality control system that maintains the protein homeostasis. When subjected to abiotic stresses, such as heat and drought, the earliest response is inhibition of protein synthesis and increase in protein folding and processing. Recently a new *Arabidopsis* cargo receptor was identified and was named TRYPTOPHAN-RICH SENSORY PROTEIN (TSPO). It is a multi-stress regulator that is transiently induced by abiotic stress and is originally described as a heme-binding protein, which interacts with ATG8 to enable the degradation of porphyrins *via* an autophagy-dependent degradation mechanism (Vanhee et al., 2011). Moreover, TSPO interacts intracellularly with the plasma membrane aquaporin PIP2;7 (PLASMA MEMBRANE INTRINSIC PROTEIN 2;7) and downregulates it in the cell. The coexpression of TSPO and PIP2;7 led to decreased levels of PIP2;7 in the plasma membrane and abolished the membrane water permeability mediated by the overexpression of PIP2;7 in transgenic seedlings. Furthermore, ABA treatment activates TSPO and triggers the degradation of PIP2;7 through the autophagic pathway. These findings suggest that TSPO acts as a selective plant-specific autophagy cargo receptor during abiotic stress (Hachez et al., 2014). Remarkably, the autophagy-mediated reduction in the quantity of PIP2;7 modulates the osmotic water permeability of membranes, which is important during heat and drought stress (Figure 1).

## ATI1, a stress-associated protein assembles into different types of novel bodies associated with either ER or plastids

With the aim of elucidating the biological processes in plants that are associated with selective autophagy in plants, our lab identified a number of plant-specific ATG8f binding proteins based on a yeast two-hybrid analysis. One of these proteins, named “Autophagy Interacting Protein 1” (ATI1), was further subjected to detailed studies. When grown under regular, non-stress conditions, ATI1 was partially associated with the endoplasmic reticulum(ER) membrane. Furthermore, upon exposure of the plants to carbon or nitrogen starvation, ATI1 was assembled into two different types of novel bodies that were associated with either the ER or the plastids (Honig et al., 2012).

When the plants were exposed to carbon or nitrogen starvation, ATI1 was incorporated into novel bodies that were either moved along the ER network, or localized inside the plastids. These novel bodies were then transported into the central vacuole in which their contents were apparently being turned over inside the plastids (Figure 1). Interestingly, the seedlings of the over-expressing ATI1 plants germinated faster and showed increased tolerance to carbon starvation and salt stress, whereas the plants with suppressed expression of ATI1 showed reduced tolerance to carbon starvation and salt stress, indicating that the biological processes using ATI1 confer faster growth and increased stress tolerance to the germinating seedlings (Michaeli et al., 2014).

The above results imply the ATI1 is a multifunctional protein, which is associated with ER-to-vacuole and plastid-to-vacuole trafficking by ATG8-mediated selective autophagy. Moreover, ATI1 is also involved in an autophagic system that promotes the seedling organization under abiotic stress conditions.

## Concluding remarks

Despite the identification of three potential cargo receptors in plants, a detailed understanding of these cargos is imperative. The questions that need to be addressed are whether there are links between the different cargo-mediated autophagy pathways, the mechanisms for the recognition and delivery of the misfolded and damaged proteins and organelles, and how the cargos are trafficked. We believe that with an in-depth research on the involvement of autophagy in abiotic stress, particularly in crop plants, it is possible to open new avenues for the enhancement of stress tolerance by using genetic and/or genetic engineering approaches, ultimately leading to enhanced production.

## Author contributions

WW and GG conceived and designed the project, and wrote the manuscript. MX and GW helped with figure revision. The manuscript was approved by all other authors.

### Conflict of interest statement

The authors declare that the research was conducted in the absence of any commercial or financial relationships that could be construed as a potential conflict of interest.
